# Supplementary material for: Spindle tubulin and MTOC asymmetries may explain meiotic drive in oocytes
Source: Nat Commun. 2018 Jul 27;9:2952. doi: 10.1038/s41467-018-05338-7 (PMC6063951; doi:10.1038/s41467-018-05338-7)
Supplement: Supplementary file 1 — Supplementary Information [file 41467_2018_5338_MOESM1_ESM.docx]

**Spindle tubulin and MTOC asymmetries may explain**

**meiotic drive in oocytes**

***Wu et al***

**Supplementary Figures**

**
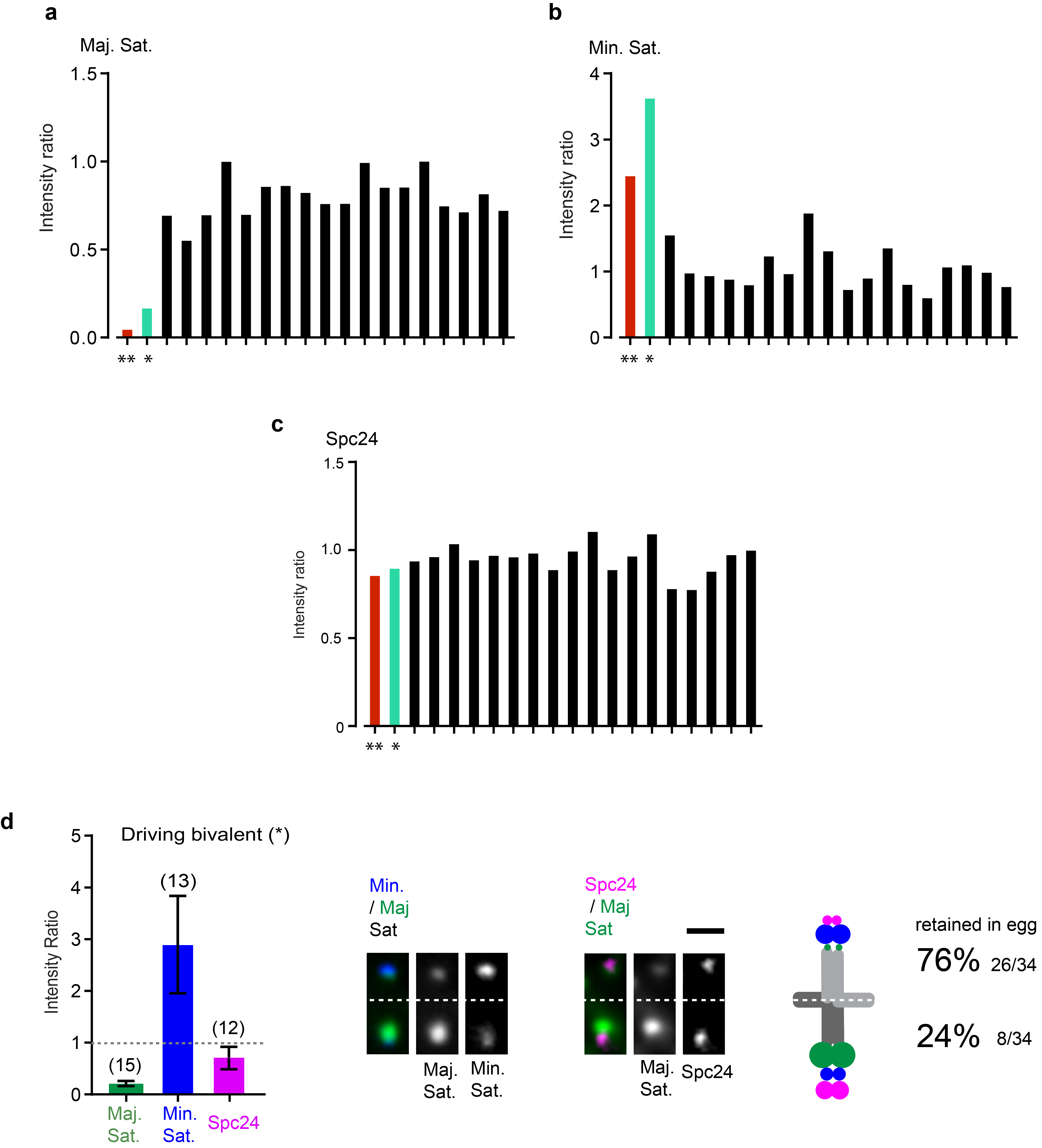
**

**Supplementary Figure 1. Measurement of satellite regions and Spc24 in bivalents of F_1_ hybrid oocytes.** (**a,b,c**) Intensity ratios for (a) the major satellite (Maj.Sat.-mClover), (b) the minor satellite (Min.Sat.-mRuby), and, (c) Spc24 (Spc24-mCherry) for all bivalents. The two bivalents identified as driving are labelled *, and ** (see Fig 1). Within each bivalent the signal associated with the larger of the two Maj.Sat. signals is the denominator and the other signal the numerator. **(d)** Intensity ratios for major satellite (green), minor satellite (blue) and Spc24 (magenta) signals on the larger driving bivalent, identified by * in (a,b,c). For each measurement, the bivalent half containing the larger major satellite repeat was the denominator. Representative images of these three regions are shown, oriented with the smaller major satellite repeat above the dotted line. The schematic displays the relative sizes of the major and minor satellites and Spc24 for each half of the bivalent, and the associated percentage retention in the egg. In parenthesis, number of bivalents examined, combined from 3 independent experiments. Error bars are 95% confidence intervals.

**
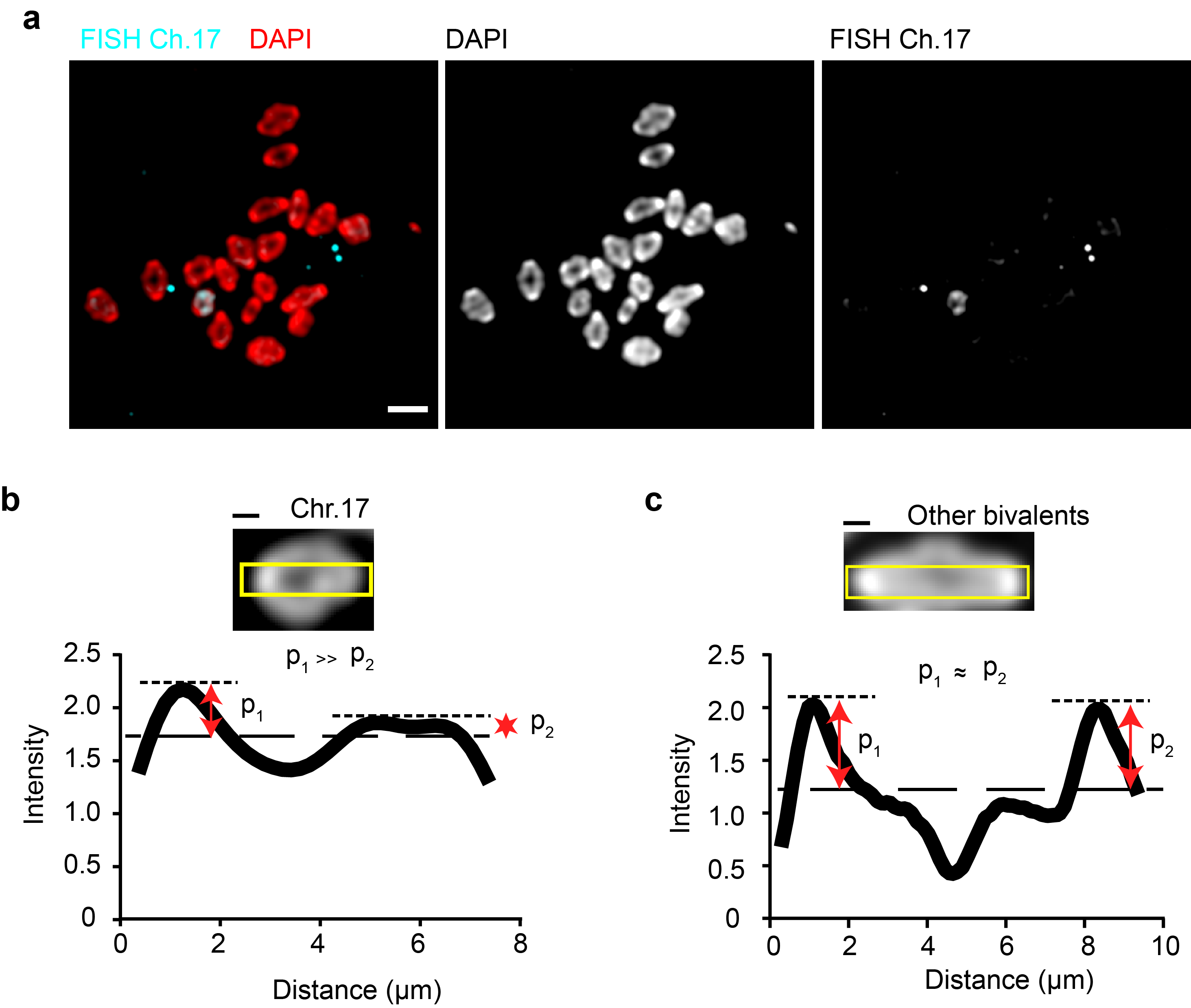
**

**Supplementary Figure 2**. **Detection of centromere asymmetry in chromosome 17 of C57BL6/SJL F1 hybrid oocytes.** (**a**) Representative images of chromosome spreads, labelled with a FISH probe for chromosome 17 (cyan) and counterstained with DAPI (red). Scale bar, 10 µm. (**b,c**) Representative image of chromosome 17 (b) and another chromosome (c) DNA stained with DAPI (grey, left) and matching intensity profile (right, corresponding to the yellow boxes). Peak intensity at centromeric regions are shown as p_1_ and p_2_ (red arrows), where the more intense peak is denoted as p_1_. Dashed lines show the average intensity in the yellow boxes. Scale bars represent 2µm.


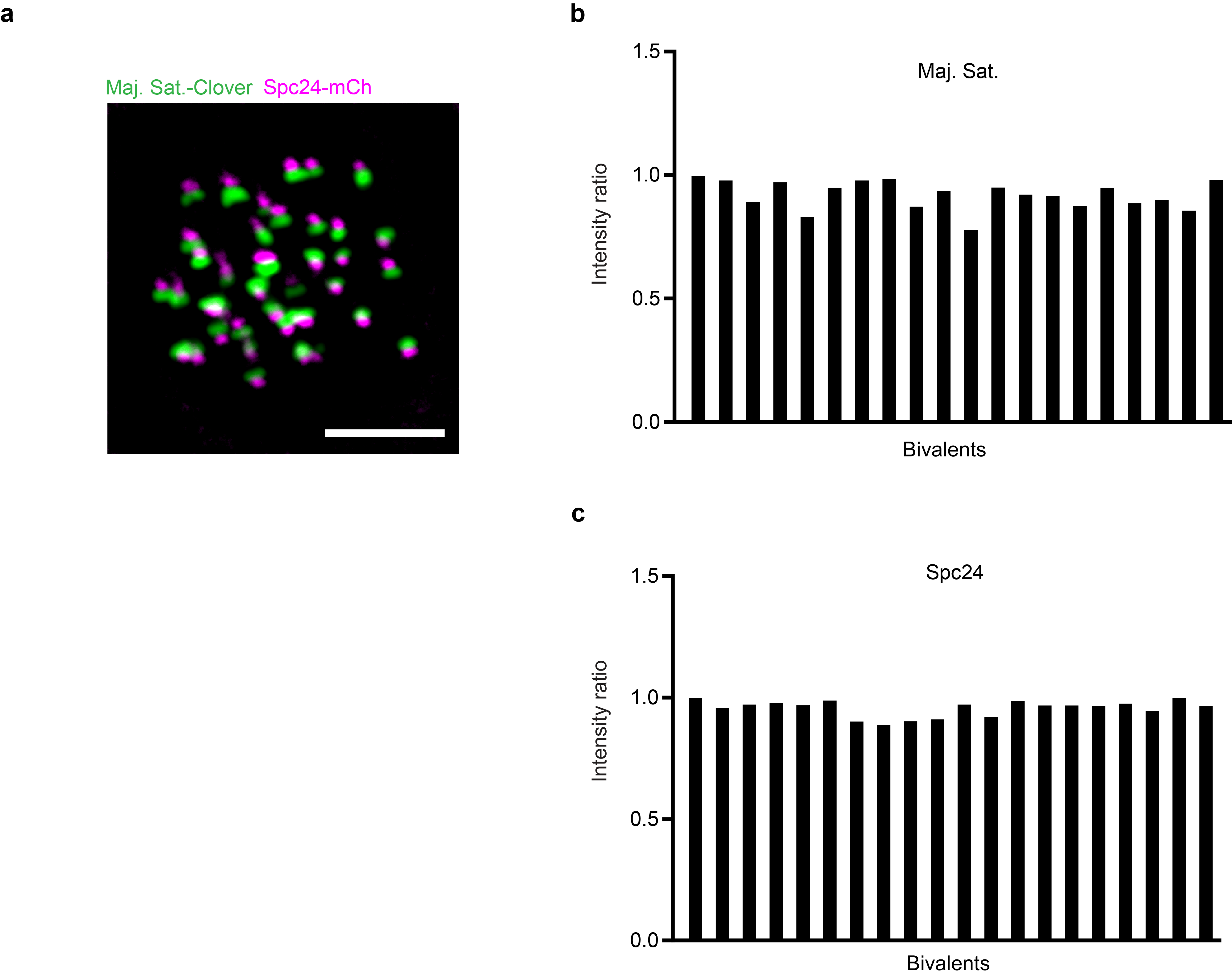


**Supplementary Figure 3. Major satellite region and Spc24 measurements in bivalents of C57Bl/6 mice.** (**a)** A representative image of bivalents expressing Maj.Sat.-mClover (green) and Spc24-mCherry (magenta) in oocytes of C57Bl/6 mice. Scale bar, 5µm. (**b,c**) Ratios for (b) major satellite (Maj.Sat.-mClover) and (c) Spc24 (Spc24-mCherry) for all bivalents. For each bivalent the signal associated with the larger of the two Maj.Sat. signals is used as the denominator.

**
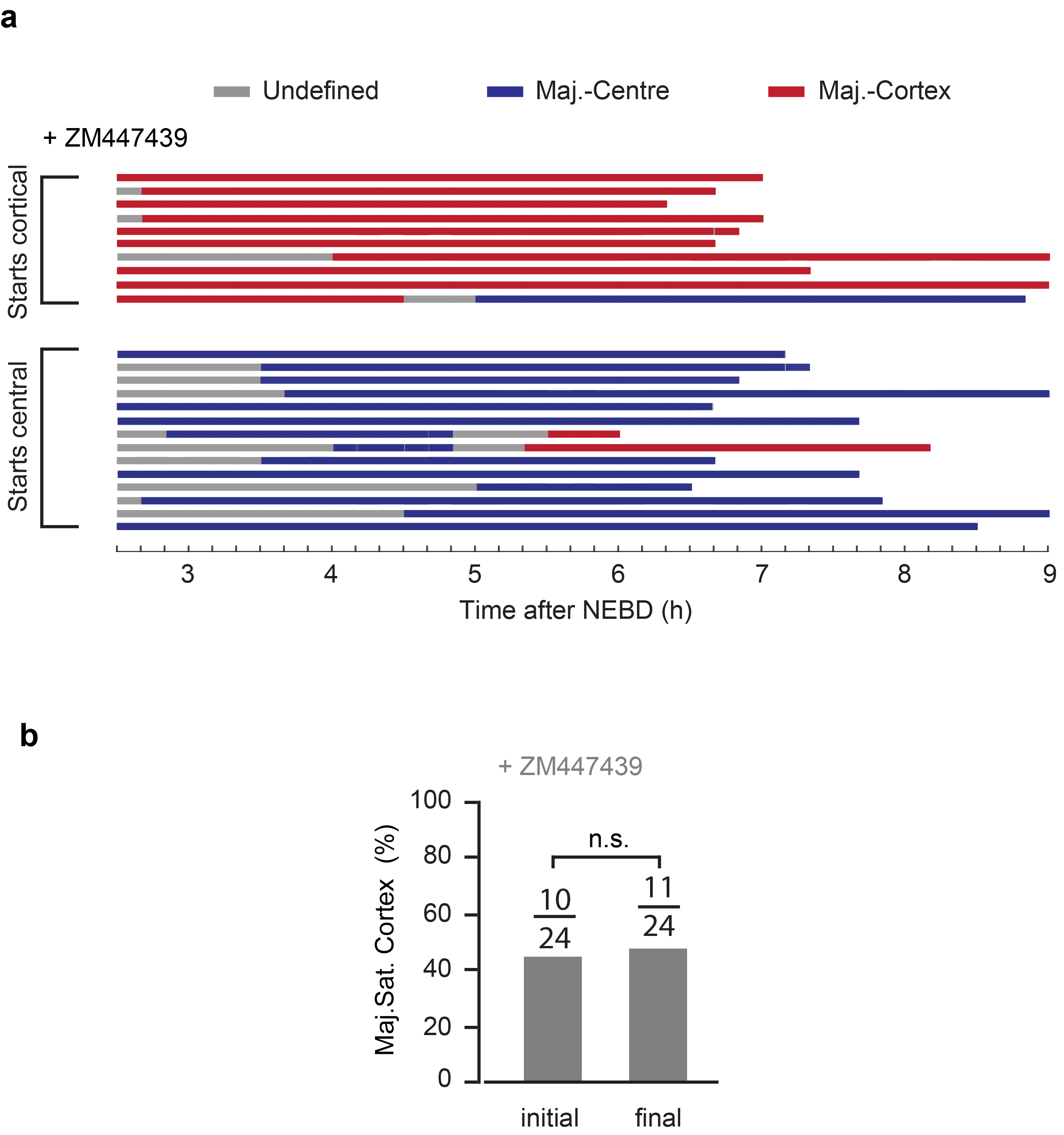
**

**Supplementary Figure 4. Meiotic drive prevented by aurora kinase inhibitor ZM447439.** (**a**) Time course of driving bivalent orientation (Maj.Sat. Cortex, red; Maj.Sat. Centre, blue; undefined, grey) from NEBD to anaphase or 9 h after NEBD in the presence of ZM447439. (**b**) Initial and final bivalent orientation, measured at the time of first bi-orientation (initial), and the frame immediately before anaphase (final) in oocytes treated with the pan-aurora kinase inhibitor ZM447439. n.s, Fisher’s exact test*.* Numbers of bivalents examined are given from 2 independent experiments


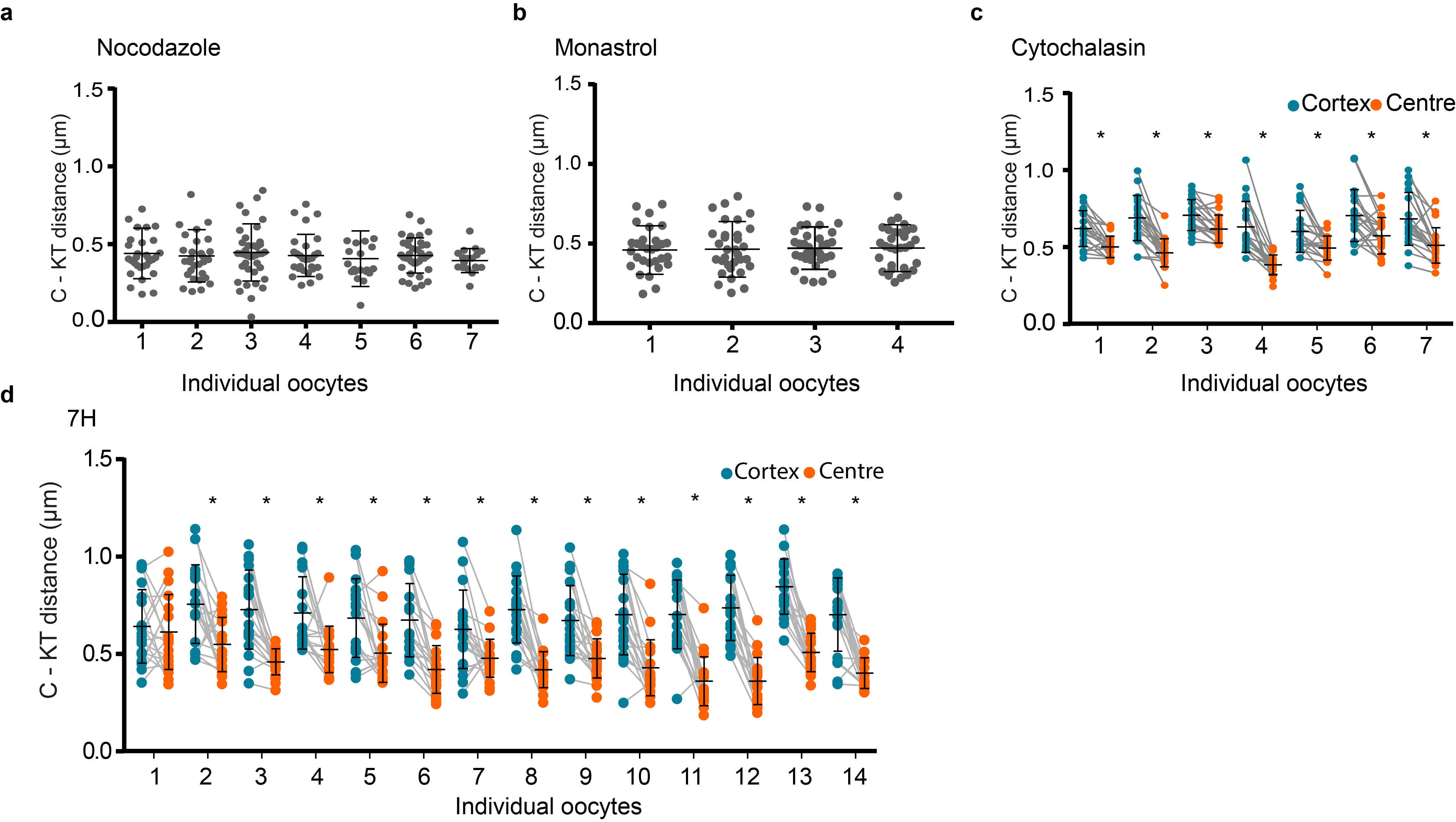


**Supplementary Figure 5. Centromere-kinetochore stretch is greater on the cortical side of bivalents.** (**a-d**) Oocytes expressing Maj.Sat.-mClover and Spc24-mCherry were matured to 4 (a,b) or 7 (c,d) hours after NEBD and treated with either, nocodazole (a), monastrol (b), or cytochalasin B (c) and centromere-kinetochore (C–KT) distances measured. Where bivalent tension was evident the side of the bivalent facing the oocyte cortex or centre (blue or orange respectively) were measured in pairs (c,d), otherwise all measures are combined (a,b). (c,d, * p<0.05, paired t-test). The number of oocytes used is indicated, with oocytes pooled from 3 (a), 2 (b), 3 (c) and 3 (d) independent repeats.
