# Supplementary material for: Spindle tubulin and MTOC asymmetries may explain meiotic drive in oocytes
Source: Nat Commun. 2018 Jul 27;9:2952. doi: 10.1038/s41467-018-05338-7 (PMC6063951; doi:10.1038/s41467-018-05338-7)
Supplement: Supplementary file 3 — Description of Additional Supplementary Files [file 41467_2018_5338_MOESM3_ESM.doc]

**Description of Additional Supplementary Files**

**File Name: Supplementary Movie 1**

**Description:**

Asymmetric bivalent tracking in meiosis I. Bivalent 17 (**, yellow box) and presumptive bivalent 4 (*, yellow circle), which we define as the two ‘driving bivalents’, were tracked from 3h after NEBD (3:00 h:min), through anaphase (8:10 h:min), and first polar body extrusion (8:20 h:min; illustrated in brightfield inset). Movie illustrates the rotation of bivalent 17 between 03:00- 03:30 h:mins, and bivalent 4 between 03:30- 04:10 h:mins. Red, chromatin; cyan, major satellite repeat. Scale bar, 5 µm.

**File Name: Supplementary Movie 2**

**Description:**

MTOC fragmentation and separation during MI. Behaviour of MTOCs from GV-stage to MII, over a 15 h time-lapse window. Red: MTOCs Cyan: chromatin.

**File Name: Supplementary Movie 3**

**Description:**

MTOC volume is greater in the cortex than the centre of the oocyte. 3D-model reconstruction shows the different MTOCs volume at cortical and central pole of spindle. Red: MTOCs Cyan: chromatin.
